# Novel SLC18A2 Variant in Infantile Dystonia-Parkinsonism Type 2

**DOI:** 10.1155/2024/4767647

**Published:** 2024-04-30

**Authors:** Sakari Kaasalainen, Harri Arikka, Mika H. Martikainen, Valtteri Kaasinen

**Affiliations:** ^1^Clinical Neurosciences, University of Turku, Turku, Finland; ^2^Department of Pediatric Neurology, Turku University Hospital, Turku, Finland; ^3^Neurocenter, Turku University Hospital, Turku, Finland; ^4^Research Unit of Clinical Neuroscience, Neurology, University of Oulu, Oulu, Finland; ^5^Department of Neurology and Medical Research Center, Oulu University Hospital, Oulu, Finland

## Abstract

Infantile dystonia-parkinsonism type 2 (PKDYS2) is a rare inherited autosomal recessive movement disorder with onset in infancy. The disease is associated with a mutation in the solute carrier family 18 member A2 gene (*SLC18A2*). There are reports of trials with dopaminergic drugs and the condition of patients given levodopa almost always worsens and dopamine agonists give varying degrees of benefit to some. Here, we report a PKDYS2 patient with a new variant in the *SLC18A2* gene who underwent multiple trials of pharmacotherapy. The abnormalities in development and neurological examination of the case were first noted at the age of 2 months, and after a series of treatment attempts (e.g., with antiepileptics) and diagnostic procedures, the diagnosis of PKDYS2 was determined when whole exome sequencing (WES) at age 6, revealed a homozygous pathologic variant NM_003054.4:c.1107dup, p.(Val370Serfs^*∗*^91) in the *SLC18A2* gene. The patient then received treatment with multiple dopaminergic drugs (e.g., levodopa, pramipexole, and methylphenidate). The patient with PKDYS2 harbored a new variant in *SLC18A2.* The phenotype of the patient resembles that of some previously reported patients with PKDYS2. The patient received minor benefits from certain dopaminergic drugs, such as pramipexole, but side effects led to the discontinuation of tested medications.

## 1. Introduction

Infantile dystonia-parkinsonism type 2 (PKDYS2; OMIM 618049) is a rare inherited autosomal recessive movement disorder with onset in infancy. The disease was described in 2013 in eight children of an extended consanguineous Saudi Arabian family with similar clinical symptoms [1]. PKDYS2 is clinically characterized by variable and complex monoaminergic signs and symptoms, including dystonia, parkinsonism, oculogyric crises (dopaminergic defect), sleep and mood disorders (serotonergic defect), excessive sweating, temperature instability, ptosis, and postural hypotension (adrenergic and noradrenergic defects) [1]. The disease is associated with a mutation in the solute carrier family 18 member A2 gene (*SLC18A2*), and the transmembrane protein encoded by *SLC18A2*, vesicular monoamine transporter 2 (VMAT2), is an ATP-dependent monoamine transporter [1]. Recently, 19 different homozygous *SLC18A2* variants were identified in 27 unrelated families involving 42 affected individuals [2]. To date, there are 58 reported cases of PKDYS2 [1–5].

As PKDYS2 is caused by a defective function of VMAT2 and dopaminergic neurotransmission, the logical conclusion has been to trial medications that affect monoaminergic function and VMAT2. Treatment with dopamine receptor agonists, such as pramipexole, has been reported to induce beneficial effects in PKDYS2 patients, particularly in those harboring the p.Pro387Leu variant [1], whereas levodopa appears to be either ineffective or worsens the symptoms of PKDYS2 [2]. However, in the largest published sample of PKDYS2 patients with various *SLC18A2* variants, pramipexole treatment 0.01-0.02 mg/kg/day was associated with only a mild improvement in 18 of 21 treated cases [2]. Here, we report a new variant in *SLC18A2* resulting in PKDYS2 clinically unresponsive to multiple dopaminergic drugs. A previous version of the manuscript has been presented as a thesis [6].

## 2. Materials and Methods

The patient was an 8-year-old male of Finnish descent. His birth was normal. The first symptoms were observed at the age of two months, as he was unable to form or maintain eye contact and his eyes involuntarily deviated upward (oculogyric crisis). Muscle tone was hypotonic. At the age of 4–12 months, his psychomotor development had not progressed normally, and he started to have generalized dystonic episodes several times per week lasting approximately 2.5 hours. The patient would also occasionally develop opisthotonus and tilt to lie on his side during the episodes with dystonic postures in the arms. Before the diagnosis of PKDYS2, the episodes were clinically interpreted as tonic epileptic seizures.

### 2.1. Investigations

Brain MRI was performed at ages 2, 8, and 10 months and 2 years with normal findings. Electroencephalography (EEG) (at age 5 months) showed no epileptiformic activity. Electromyography and nerve conduction studies (ENMG) (at age 9 months) were also normal. Cerebrospinal fluid (CSF) analysis was normal (leucocyte and erythrocyte counts, glucose, protein, and lactate concentrations). In urine amino acid analysis, cysteine, lysine, and beta-alanine were excreted in the urine in excess, and glutaminic acid was excreted in smaller concentrations than normal. Analysis of white blood cell-derived mitochondrial DNA did not reveal the common point mutations m.3243A > G, m.8344A > G, or m.8993T > C/G. Whole exome sequencing (WES) at age 6 revealed a homozygous NM_003054.4:c.1107dup, p.(Val370Serfs^*∗*^91) variant in the *SLC18A2* gene (SNP rs754623209). The variant creates a shift in the reading frame and results in a premature stop codon. According to the American College of Medical Genetics and Genomics (ACMG) classification [7], the variant is likely pathogenic (class 2), and it is very infrequent in databases (gnomAD 0.000043, 1000 Genomes 0.000045, and CentoMD 0.000067). In addition to the pathogenic *SLC18A2* variant in WES, the identification of an additional variant in the *ATP7A* gene initially raised concerns about Menkes disease, given its association with copper deficiency. To investigate this possibility, an assessment of copper and ceruloplasmin concentrations was conducted using both blood and skin fibroblast samples. Despite the presence of the *ATP7A* gene variant, the findings revealed normal levels of copper and ceruloplasmin, excluding the copper metabolism abnormality associated with Menkes disease in association with the clinical phenotype not concordant with Menkes syndrome.

## 3. Results

Several drug treatments were tried over the disease course. Before the genetic diagnosis was confirmed at the age of 2 years, valproic acid (300 mg/day) was tested because of suspected epilepsy. Valproate only mildly reduced the duration of dystonic episodes, and the drug was discontinued after 6 months due to limited efficacy. At age 2.5 years, levodopa-carbidopa (75 mg/day) showed mixed effects, as it increased the patient's ability to control his head movements but caused hyperactive behavior and insomnia. Due to the side effects, levodopa was discontinued during the first month of the trial. After the genetic diagnosis was established, pramipexole was initiated at a dose of 0.18 mg/day, and after one month, the patient was able to support his head better, swallowing and breathing improved, and the number of dystonic episodes reduced. However, during the following months, the efficacy of pramipexole waned, although the dose was increased to 0.36 mg/day, while the side effects became more prominent. Side effects included nausea and dysphoria, slowing down of bowel movements, dyskinesias, and problems with sleep and general restlessness. Therefore, due to limited efficacy and side effects, the dose of pramipexole was reduced and eventually the medication was discontinued after 9 months of treatment. In addition, amantadine (100 mg/day, at age 7.5 years) was tested without beneficial effects, followed by methylphenidate (5 mg/day, age 8 years), which induced both positive and negative effects as the patient's ability to support his head and alertness improved, but the number of dystonic episodes increased, leading to discontinuation after 4 months of treatment.

Nonpharmaceutical care for the patient was organized by a team consisting of a physician, dietician, physiotherapist, occupational therapist, and rehabilitation instructor. The patient received regular weekly physiotherapy from the age of 2 months onward, and occupational therapy was arranged for special arrangements in daycare and schooling.

At the age of 9 years, the patient was severely disabled and required assistance in all aspects of life. He expressed himself by making sounds, gesticulating, smiling, and crying. The phenotype consisted of global developmental delay, severe speech impairment, a generally hypotonic musculature, and abnormal motor function, including hyperkinetic movements, oculogyric crises, and severe difficulty in completing voluntary movements. He also continued to have episodes of increased tonic muscle contractions, clenching fists, and upward deviation of the eyes together with trunk dystonia and hyperhidrosis. The patient had these episodes approximately once every four days, and he remained conscious throughout.

## 4. Discussion

Here, we describe the first Finnish patient with PKDYS2 with a new variant c.1107dup, p.(Val370Serfs^*∗*^91). No functional analyses for the detected *SLC18A2* variant were performed. This variant was deemed pathogenic based on the resulting premature stop codon, ACMG classification as likely pathogenic, an extreme rarity in large databases, and the clinical phenotype in line with those previously described in patients with PKDYS2. The phenotype of the patient can be described as a complex, severe movement disorder with muscular hypotonia and dystonic episodes as prominent features. Our patient did not receive clear clinical benefits from valproate, levodopa, or amantadine. However, small/moderate doses of pramipexole and methylphenidate were associated with mild, but transient improvements in the patients' ability to support his head better, swallowing, and breathing, but the drugs had to be discontinued due to prominent side effects. The phenotype and poor treatment efficacy seem roughly comparable with previous reports of *SLC18A2* variants c.710C > A, c.1160C > T, c.926C > T, c.181C > T, c.216dupA, and c.282delA [2–4]. It should be noted that even among individuals with the same variant, the treatment results have varied. For example, some patients with the variant c.710C > A have received clear benefits from dopamine receptor agonists, while others have had much milder beneficial and somewhat questionable effects [1, 2].

The patient reported here initially responded to pramipexole, but severe side effects limited its usefulness. This contrasts with some previous studies reporting that pramipexole was associated with clear improvements without clinically relevant side effects [1]. However, on the basis of the largest reported PKDYS2 cohort to date [2], the initial interpretation of an excellent pramipexole response might have been due to the more benign phenotype in PKDYS2 patients with certain variants. Overall, it seems there is a considerable interindividual variation in response to pramipexole in PKDYS2, which could be driven by phenotypical differences and baseline differences in dopamine receptor density or affinity. It is possible that pramipexole could be beneficial without major side effects in earlier phases of the disease when receptor function is sufficient for effective dopamine agonist treatment. We are not aware of previous reports of the effect of methylphenidate on PKDYS2. Methylphenidate is used for attention-deficit hyperactivity disorder (ADHD) in both children and adults, and it blocks the reuptake of norepinephrine and dopamine in presynaptic neurons, leading to increased concentrations of dopamine and norepinephrine in the synaptic cleft. Due to this methylphenidate-induced increase in monoaminergic function, we hypothesized that it could also improve neurotransmission in PKDYS2 and provide improvement in motor function. Our patient did benefit from methylphenidate to some extent, but clinically relevant side effects eventually led to the discontinuation of the drug. Therefore, we propose that, in individual cases, methylphenidate could be one of the tested drugs for patients who are unresponsive to the other classes of medications used for PKDYS2.

The phenotype of our patient resembles some PKDYS2 patients reported in earlier studies [2–4]. In particular global developmental delays in nonambulation and communication, truncal hypotonia, dystonic episodes, parkinsonism, temperature instability, and oculogyric crises are prominent clinical features also seen in our patient. However, our patient did not have epileptic seizures or epileptiform activity in EEG, which are reported in some PKDYS2 families [2]. In conclusion, PKDYS2 with the variant c.1107dup, p.(Val370Serfs^*∗*^91) causes a clinical phenotype of a complex movement disorder. Treatments with pramipexole and methylphenidate induced some positive symptomatic effects, but side effects limited their further use. In order to enhance our understanding of PKDYS2 and its implications, future research directions may involve the execution of larger cohort studies, despite the rarity of patients with this condition. In addition, conducting functional assays using relevant models could provide insights into the mechanistic aspects associated with PKDYS2. Furthermore, the exploration of animal models harboring similar variants could serve as an invaluable tool to dissect the pathophysiology of this genetic variation. These potential avenues for further investigation would advance our comprehension of PKDYS2 and its clinical implications.

## Data Availability

The datasets for this article are not publicly available due to concerns regarding participant/patient anonymity. Requests to access the datasets should be directed to the corresponding author.
